# Having your cake and eating it - *Staphylococcus aureus* small colony variants can evolve faster growth rate without losing their antibiotic resistance

**DOI:** 10.15698/mic2017.08.587

**Published:** 2017-08-01

**Authors:** Gerrit Brandis, Sha Cao, Douglas L. Huseby, Diarmaid Hughes

**Affiliations:** 1 Department of Medical Biochemistry and Microbiology, Biomedical Center, Uppsala University, Uppsala, Sweden.

**Keywords:** SCV (small colony variant), suppressors, SrrAB, tRNA mutations, intragenic suppressor, extragenic suppressor, ATP generation

## Abstract

*Staphylococcus aureus* can produce small colony variants (SCVs) during infections. These cause significant clinical problems because they are difficult to detect in standard microbiological screening and are associated with persistent infections. The major causes of the SCV phenotype are mutations that inhibit respiration by inactivation of genes of the menadione or hemin biosynthesis pathways. This reduces the production of ATP required to support fast growth. Importantly, it also decreases cross-membrane potential in SCVs, resulting in decreased uptake of cationic compounds, with reduced susceptibility to aminoglycoside antibiotics as a consequence. Because SCVs are slow-growing (mutations in *men* genes are associated with growth rates in rich medium ~30% of the wild-type growth rate) bacterial cultures are very susceptible to rapid takeover by faster-growing mutants (revertants or suppressors). In the case of reversion, the resulting fast growth is obviously associated with the loss of antibiotic resistance. However, direct reversion is relatively rare due to the very small genetic target size for such mutations. We explored the phenotypic consequences of SCVs evolving faster growth by routes other than direct reversion, and in particular whether any of those routes allowed for the maintenance of antibiotic resistance. In a recent paper (mBio 8: e00358-17) we demonstrated the existence of several different routes of SCV evolution to faster growth, one of which maintained the antibiotic resistance phenotype. This discovery suggests that SCVs might be more adaptable and problematic that previously thought. They are capable of surviving as a slow-growing persistent form, before evolving into a significantly faster-growing form without sacrificing their antibiotic resistance phenotype.

The increasing prevalence of antibiotic-resistant bacteria is causing a global healthcare problem with significantly restricted therapeutic options and increased risks of morbidity and mortality associated with common bacterial infections. Understanding the processes and evolutionary trajectories associated with resistance development could be valuable in aiding the development and optimized use of new generations of novel antibiotics. On the one hand, there is a basic science interest in understanding how bacteria can respond and evolve in the face of antibiotic challenge. On the other hand, there is also an interest from both the pharmaceutical industry and healthcare policy makers in applying knowledge of resistance evolution to guide the discovery, development, and use of new generations of novel antimicrobial drugs with the aim of reducing the probability of rapid resistance development.

In studying evolution one frequently sees evidence of trade-offs such as a reduction in relative bacterial fitness associated with mutations reducing susceptibility to antibiotics. In the example of *S. aureus* SCVs, the cost of high-level resistance to aminoglycosides is a very large reduction in growth rate. Because of their very slow growth, populations of SCVs can be rapidly overgrown by any mutant bacteria in the population that reverts the original mutation, or that acquires a second-site intragenic suppressor mutation restoring activity to the mutated gene. In some of the earlier literature this high frequency phenotypic instability was taken as evidence that SCVs were reversible phenotypic variants rather than stable genetic variants. The current evidence is that most, and probably all, of the classically defined *S. aureus* SCVs (those displaying hemin or menadione auxotrophy), are due to mutations affecting pathways important for respiration.

An advantage enjoyed by SCVs is that they combine the ability to survive in the human body (forming persistent infections that survive drug therapy) with the propensity to frequently generate fast-growing offspring (that test the current environment) and when the conditions are right, can re-establish a major infection. Previous genetic knowledge about *S. aureus* SCVs indicated that the phenotype could be caused by mutations in many different genes of either the menadione or hemin biosynthesis pathways (including *menA, B, E,* and* hemB, C, D, E, H*). Some of the mutations involved were single amino acid substitutions but the fact that many were frameshift or nonsense mutations indicated that the critical feature connecting genotype with the SCV phenotype was that the SCV-generating mutation should knock out a critical enzyme in either pathway. The genetic data is compatible with previous experimental observations that many SCVs have the ability to generate fast-growing offspring, either by reversion or by the occurrence of intragenic suppressor mutations, that restore respiration at the cost of losing antibiotic resistance (Figure 1). However, until recently (mBio 8: e00358-17), it was not known whether SCVs had alternative evolutionary pathways to increase their growth rate, including pathways that did not involve loss of antibiotic resistance.

**Figure 1:**
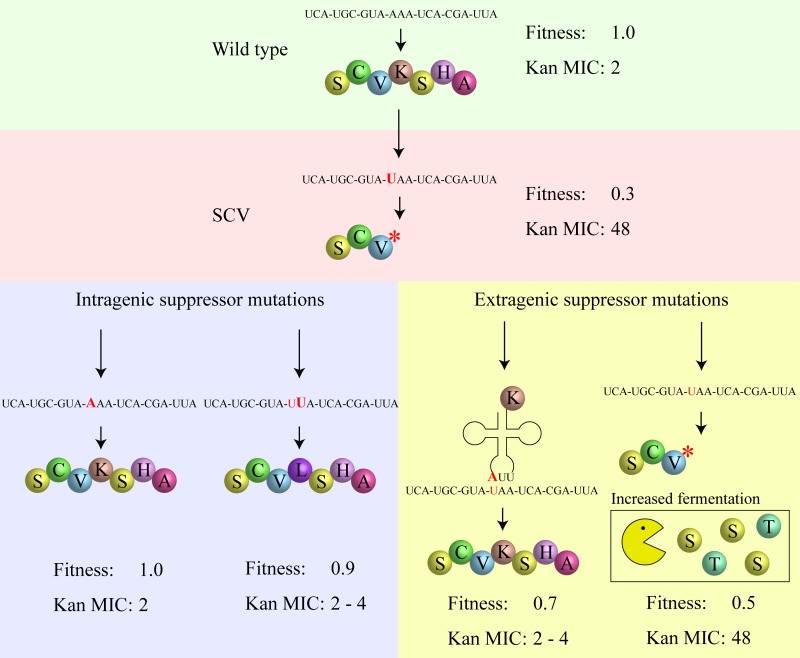
FIGURE 1: Evolutionary trajectories to suppress an SCV phenotype. A hypothetical wild-type nucleotide and protein sequence associated with high relative fitness and low MIC for the aminoglycoside kanamycin is shown. In the SCV mutant shown there is an internal stop codon in gene involved in respiration (*hem* or *men*), leading to decreased fitness and increased kanamycin MIC. Suppression can occur either by intragenic mutation (direct reversion or a second-site intragenic mutation) or by extragenic mutation (for example, a translational suppressor mutation, or a mutation in *srrAB*). Translational suppression causes production of a pseudo-wild-type protein, resulting in increased fitness with reduced aminoglycoside resistance. In contrast, mutations that constitutively activate the SrrAB two-component system increase growth rate by increasing the production of ATP by fermentation pathways, without reducing resistance to aminoglycosides.

We initially selected for faster growth among multiple independent cultures of 8 different SCVs, each carrying a missense, nonsense or frameshift mutation in a *hem* or *men* gene. For 4/8 of these SCVs all of the fast growers selected were caused either by reversion or by intragenic suppression of the SCV mutation. However, in the remaining 4 SCVs, many of the independently selected fast growers (*hemH* UAA, 11/20; *menA* UGA, 3/10; *menB* D151N, 8/34; and *menE* UAA, 16/22) had acquired extragenic suppressor mutations (Figure 2). The majority of these extragenic suppressors (32/38) were mutations in genes affecting protein synthesis (tRNA genes, ribosomal protein S5, and RF-2) suggesting that they reverse the SCV phenotype by translational suppression. This hypothesis is supported by the relationships between specific mutant tRNAs (and mutant RF-2) and the particular SCV mutations they suppress (mBio 8: e00358-17). Translational suppression is also consistent with the loss of, or strong reduction in, the level of antibiotic resistance observed with this class of mutation.

**Figure 2:**
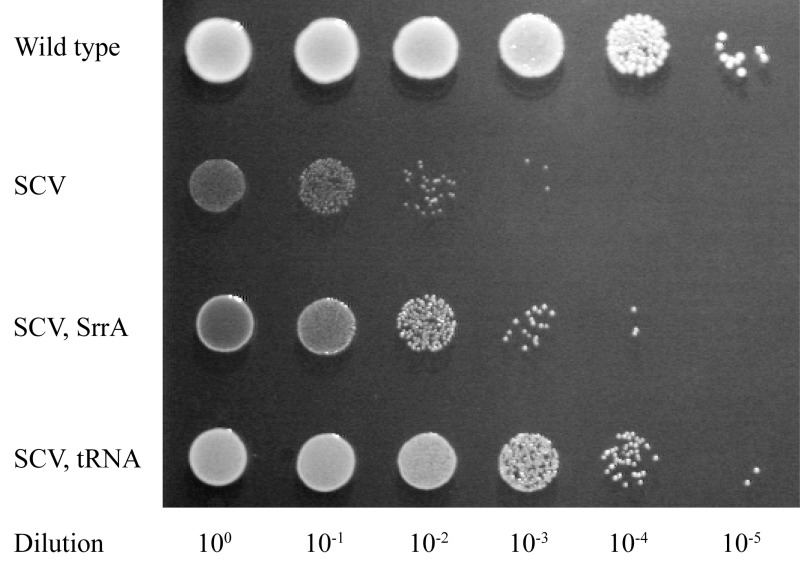
FIGURE 2: Spot test assay showing relative fitness differences between wild-type, SCV, and suppressed mutants. For each strain 10 colonies from an LB agar plate incubated at 37°C for 24 hours were suspended in 1 mL 0.9% NaCl. Each spot represents 5 μL of cell suspension pipetted onto LB agar, with 10-fold dilutions as indicated, incubated at 37°C for 24 hours before photography. The SCV mutation is *menB* D151N, and the suppressed mutants shown are isogenic strains carrying suppressor mutations in *srrA* and tRNA-Asp, respectively.

The remaining 6 extragenic suppressors (isolated as suppressors of an SCV caused by *menB* D151N) had acquired mutations in the *srrAB* genes (Figure 2), and importantly, these faster-growing strains maintained their resistance to aminoglycosides. To validate this result, and to exclude the possibility that *srrAB* suppressors were specific for only one SCV mutation, we selected suppressors for 5 additional SCVs, and screened them for mutations in *srrAB*. We identified *srrAB *mutations as suppressors in 4 of these SCVs (*menB* G121D, *menE* Y31UAA, *menE* D98G, and *hemE* A211fs) showing that this is a general and common mutational pathway for SCVs to achieve a faster growth rate. In each case the resistance to kanamycin was maintained in the faster growing mutants.

SrrAB (staphylococcal respiratory response) is a two-component transcription regulatory system in *S. aureus* that acts in the global regulation of virulence factors and contributes to its survival in anaerobic environments. The SCV-suppressor mutations were shown to work by constitutively activating the SrrAB regulatory system. In contrast to other classes of suppressor mutation (reversion, intragenic suppressors, translational suppressors) the SrrAB suppressors did not restore membrane potential, explaining their continued resistance to aminoglycosides. This however raised the question of how mutations in *srrAB* could increase growth rate if respiration was not restored. It was already known that SCVs compensate phenotypically for the loss of ATP from respiration by increasing expression of genes involved in arginine deimination and pyruvate fermentation, and use these pathways to produce ATP to support their (very slow) growth rate. Using transcriptome analysis, we could show that the SrrAB suppressors caused two significant effects: (i) they caused a further increase in the expression of the arginine deiminase and pyruvate fermentation pathways than observed in SCV mutants, and, (ii) they caused upregulation of several peptide transporters, and of enzymes involved in the fermentation of several amino acids, including serine and threonine (Figure 1). We showed that adding serine and threonine to the growth medium specifically increased the growth yield of strains with *srrAB* suppressors but not of the parental SCV mutant (mBio 8: e00358-17).

In summary, mutations in *srrAB* that constitutively activate the SrrAB two-component system increase the expression of genes involved in amino acid import and fermentation, allowing SCV mutants to increase their ATP production (and growth rate) in the absence of a functioning respiration pathway. A consequence of using this bypass mechanism to increase growth rate without restoring respiration is that *srrAB* mutants suppress the SCV phenotype while maintaining their resistance to aminoglycosides.

These *in vitro* results leave outstanding the question of the clinical significance of the *srrAB* class of suppressors. It is plausible that the same strong selective pressure for increased growth rate in the lab that gave rise to the *srrAB* suppressor mutations could also generate these mutations in a clinical setting. These mutants, if viable in the host, could present an even greater clinical problem than the original SCV, since the primary limiting feature of the SCV (the slow growth phenotype) could be partially ameliorated without sacrificing the reduced antibiotic susceptibility of the parental SCV.

